# Posterior-only correction surgery for idiopathic scoliosis Lenke type 5c: differences of strategies and outcomes between adult patients and adolescent patients

**DOI:** 10.1007/s43390-023-00647-4

**Published:** 2023-01-29

**Authors:** Xiyu Pan, Jun Qiao, Zhen Liu, Benlong Shi, Saihu Mao, Song Li, Xu Sun, Zezhang Zhu, Yong Qiu

**Affiliations:** Division of Spine Surgery, Department of Orthopedic Surgery, Nanjing Drum Tower Hospital, The Affiliated Hospital of Nanjing University Medical School, Zhongshan Road 321, Nanjing, 210008 China

**Keywords:** Lenke 5c, Adolescent idiopathic scoliosis, Adult idiopathic scoliosis, Clinical outcomes

## Abstract

**Purpose:**

To compare radiographic parameters, and functional and surgical outcomes between lumbar adolescent idiopathic scoliosis (AIS) and lumbar adult idiopathic scoliosis (AdIS).

**Methods:**

A retrospective study was performed to identify Lenke 5c type AIS and AdIS patients from our scoliosis database who had undergone posterior surgical treatment for scoliosis. Preoperative and postoperative radiographic and clinical outcomes were compared between the two groups.

**Results:**

A total of 22 patients were included in AdIS group, and 44 matched patients in AIS group. AdIS group had significantly larger L3 and L4 tilt and translation than AIS group (*P* < 0.05). AdIS group had larger T10-L2 angle and smaller T5–T12 angle (*P* < 0.05). AdIS group had higher VAS scores (*P* < 0.05) and pain domain of SRS-22 scores (*P* < 0.05) as compared to AIS group. Correlation analysis demonstrated positive relationship between VAS scores and T10-L2 angle (*r* = 0.492, *P* < 0.05). AdIS group was fused longer than AIS group (*P* < 0.05). Cobb angle of TL/L curve was larger and correction ratio was smaller at AdIS group (*P* < 0.05). AdIS group still had significantly larger L3 and L4 tilt and translation than AIS group (*P* < 0.05). CT measurements demonstrated larger postoperative vertebral body rotation at apical vertebrae and LIV at AdIS group (*P* < 0.05). Vertebral correction ratio was smaller at AdIS group (*P* < 0.05).

**Conclusion:**

Lenke 5c AdIS patients had greater preoperative and postoperative L3 and L4 tilt and translation, as well as less correction of major curve and vertebral body derotation than AIS patients. However, the incidence of adding-on was similar between the two groups.

## Introduction

Adolescent Idiopathic Scoliosis (AIS) is an abnormal curvature of the spine exceeding 10 degrees, diagnosed in adolescence and in which the etiology is unknown [1]. According to curve magnitude and maturity of the patients, the main treatment options for scoliosis include observation, bracing, and operation. For the curvature less than 25 degrees, patients can be observed on a 6- to 12-monthly basis with clinical and radiological follow-up [2]. For unmatured patients with curves between 25 and 45°, bracing should be considered [3]. Correction surgery is indicated to patients with curves beyond 45° [4]. However, curves in some patients continued to progress even after skeletal maturity, especially for those who had curves larger than 50° [5]. There are two main approaches to carry out the correction surgery, posterior and anterior, while posterior approach becomes the trend due to the safety and correction outcome. Although anterior surgery can save surgical segments, there are many vessels and organs anteriorly, which affects the safety of the surgery. In addition, back pain was more often seen in adult patients than in adolescent patients, which could be a reason for pursuing corrective surgery [5]. Several studies have compared surgical outcomes between AIS and adult idiopathic scoliosis (AdIS) [6–8]. However, most of the studies focused on patients with major thoracic curves (Lenke 1 or 2 types), and there was no study concerning patients with thoracolumbar or lumbar curves (Lenke 5 or 6 types). Different from that of thoracic vertebrae, the stress of lumbar vertebrae is greater and facet joint degeneration is more obvious for adult population [9]. In addition, the key to lumbar correction is vertebral body derotation. The intervertebral disc elasticity of adult scoliosis patients is worse than that of AIS patients, and the correction rates may be different [10]. Finally, the incidence of low back pain in patients with lumbar AdIS is higher than that in thoracic spine [11]. Whether corrective surgery can alleviate low back pain needs to be investigated. It remains to be studied whether corrective surgery for AdIS could achieve the similar radiological or life quality improvement as for AIS. With the increase of the age, intervertebral disc degeneration as well as muscle atrophy would make surgical strategy changed. This study limited the adult age group and curve magnitudes to make the groups comparable to make clear the influence of maturity other than degeneration on surgical strategy. The objectives of this study were to compare radiographic parameters, and functional and surgical outcomes between lumbar AIS and lumbar AdIS underwent posterior procedure to determine the suitable surgical time for idiopathic scoliosis.

## Materials and methods

A retrospective study was performed to identify Lenke 5c type AIS and AdIS patients from our scoliosis database who had undergone posterior surgical treatment for scoliosis between May 2006 and September 2017. Inclusion criteria for the AdIS group consisted of age between 25 and 50 years, Cobb angles of the major curve ranging from 45 to 75°, single-stage posterior-only correction and fusion surgery, and at least 2 years of postoperative follow-up. Patients who had other skeletal deformities or had a history of spine surgery or incomplete radiographic data were excluded. From May 2006, posterior approach was the only surgical option to treat scoliosis and all pedicle screw system as well as derotation technique had become standard surgical strategy at our institute. A group of AIS patients aged between 10 and 18 years with complete radiographic, surgical data, and quality-of-life score were selected and were well matched to the AdIS group at a 2:1 ratio in terms of curve pattern, sex, Cobb angle of main curve (within 5°), and length of follow-up (within 6 months). Factors other than these were not considered in the matching process.

All patients in this study had at least 2 years of postoperative follow-up. Preoperative, postoperative, and the last follow-up standing posteroanterior full spine radiographs were acquired. Radiographic parameters were evaluated for coronal and sagittal spinal alignment combined with pelvic parameter: (1) curve angles of major thoracolumbar/lumbar curves and minor thoracic curves; (2) range of thoracolumbar/lumbar curve; (3). L3 and L4 translation: distance between CSVL and vertical line across midpoint of L3 or L4 vertebrae; (4) L3 and L4 tilt: the angle between the inferior endplate of L3 or L4 and the sacrum; (5) sagittal parameters included T5–T12 angle, T10-L2 angle, L1-S1 angle, and SVA; (6) pelvic parameters included pelvic tilt, sacral slope, and pelvic incidence; (7) correction ratio: preoperative Cobb angle–postoperative Cobb angle/preoperative Cobb angle. CT three-dimensional reconstruction was performed pre- and postoperatively. Vertebral body rotation was measured at apical vertebrae and lowest instrumented vertebrae: angle formed between a perpendicular line starting from the posterior central aspect of the spinal canal and a straight line through the posterior central aspect of the spinal canal and the middle of the vertebral body. Preoperative and last follow-up VAS scores and SRS-22 scores were acquired for all the patients. Surgical complications were also recorded. Adding-on was defined as a progressive increase in the number of vertebrae included within the distal curve, with an increase of horizontal translation of LIV + 1 by more than 5 mm.

### Statistical analyses

Data were statistically analyzed using IBM SPSS Statistics 23.0 (SPSS Inc., Chicago, IL). Average values were reported as mean (SD). Summary statistics from the analyses of variance calculations were used to provide 95% confidence intervals for the error in measurements. Chi-square test was used in the comparison of the incidence of distal adding-on between the two groups. Independent sample *t* test was used in the comparison of the difference between the two groups as well as the difference between preoperative and final follow-up. Pearson correlation test was used to detect the correlation between radiographic parameters and VAS scores. The level of significance was set at *P* < 0.05.

## Results

**Fig. 1:**
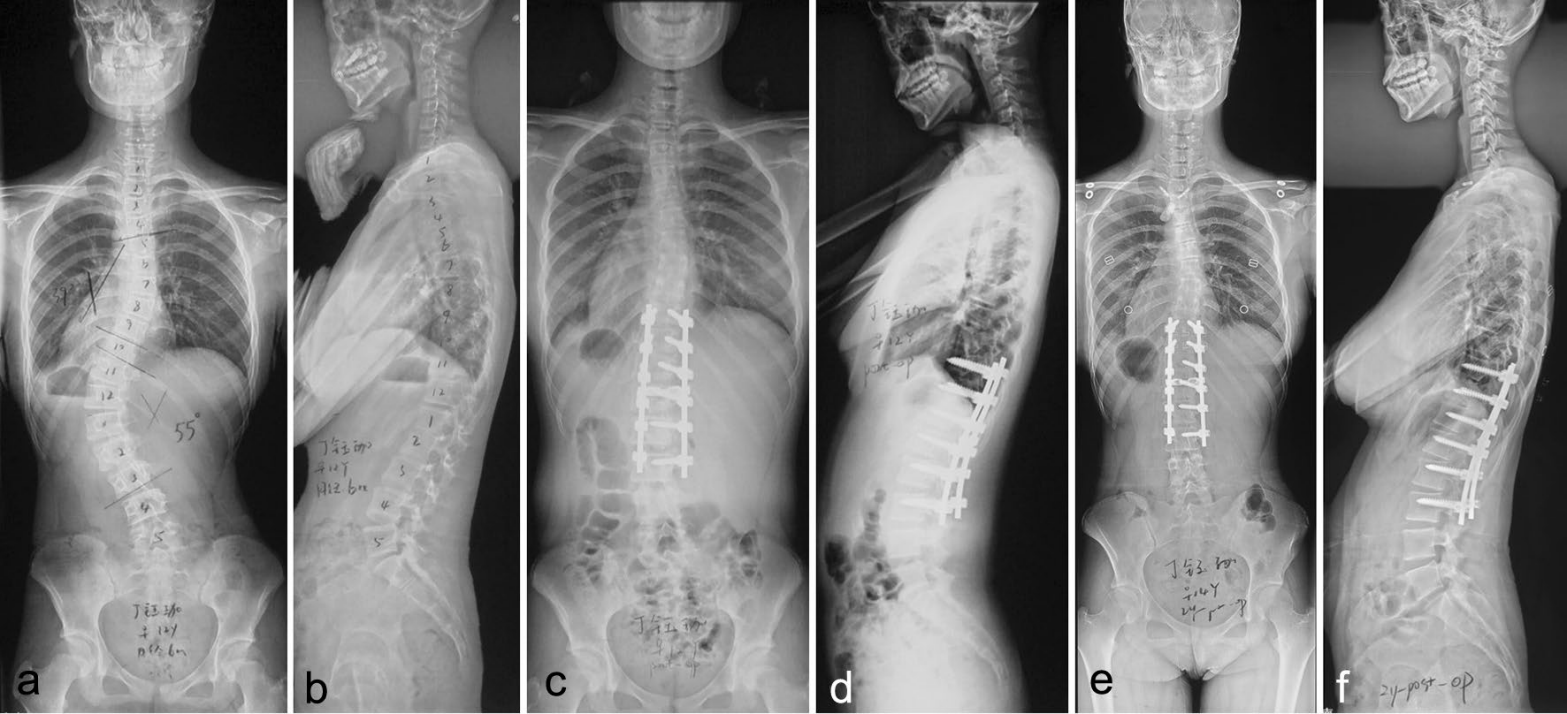
A female patient aged 12 years with 55° left thoracolumbar curve (**a**, **b**), receiving T10-L3 posterior correction surgery (**c**, **d**); 2 year postoperative radiographs did not show significant correction loss (**e**, **f**)

A total of 22 patients with an average age of 37.3 ± 8.7 years (range, 30–48 years) at the time of surgery were included in AdIS group, and 44 matched patients with an average age of 14.7 ± 3.2 years (range, 11–18 yr) at the time of surgery in AIS group. There was no difference between the two groups in terms of main thoracolumbar/lumbar Cobb angle (51.5 ± 6.4 vs. 49.2 ± 7.3°; *p* = 0.873), sex (*p* = 0.465), and curve patterns. LIV was at L3 for 8 patients, L4 for 11 patients, and L5 for 3 patients at AdIS group. At AIS group, LIV was at L3 for 28 patients and at L4 for 16 patients. AdIS group was fused longer than AIS group (7.8 ± 2.4 vs. 6.4 ± 1.6 levels; *p* < 0.05). In the regard of operation data, AdIS group had significant higher operation time (173.6 ± 32.8 vs. 234.2 ± 44.5 min; *p* < 0.001) and blood loss (578.3 ± 94.7 vs. 739.6 ± 127.3 ml; *p* < 0.001) than AIS group (Fig. 1).

As to coronal parameters, postoperative Cobb angle of TL/L curve was larger (17.4 ± 8.9 vs. 10.2 ± 7.2°; *p* = 0.009) and correction ratio was smaller (65.6 ± 15.7 vs. 79.1% ± 13.2%; *p* = 0.012) at AdIS group than AIS group. AdIS group had significantly larger preoperative L3 tilt (29.4 ± 6.4 vs. 21.7 ± 5.3°; *p* = 0.027), L4 tilt (23.7 ± 5.7 vs. 15.2 ± 4.6°; *p* = 0.017), L3 translation (33.4 ± 5.6 vs. 23.1 ± 4.2 mm; *p* = 0.012), and L4 translation (22.6 ± 5.7 vs. 14.9 ± 5.0 mm; *p* = 0.021) than AIS group. After the posterior correction surgery, AdIS group still had significantly larger L3 tilt (9.4 ± 4.1 vs. 4.2 ± 3.5°; *p* < 0.001), L4 tilt (7.2 ± 3.7 vs. 3.8 ± 3.2°; *p* < 0.001) and L3 translation (22.4 ± 4.3 VS. 15.2 ± 4.6 mm; *p* = 0.029), L4 translation (16.2 ± 3.9 vs. 11.4 ± 3.2 mm; *p* = 0.016) than AIS group at final follow-up.

**Fig. 2:**
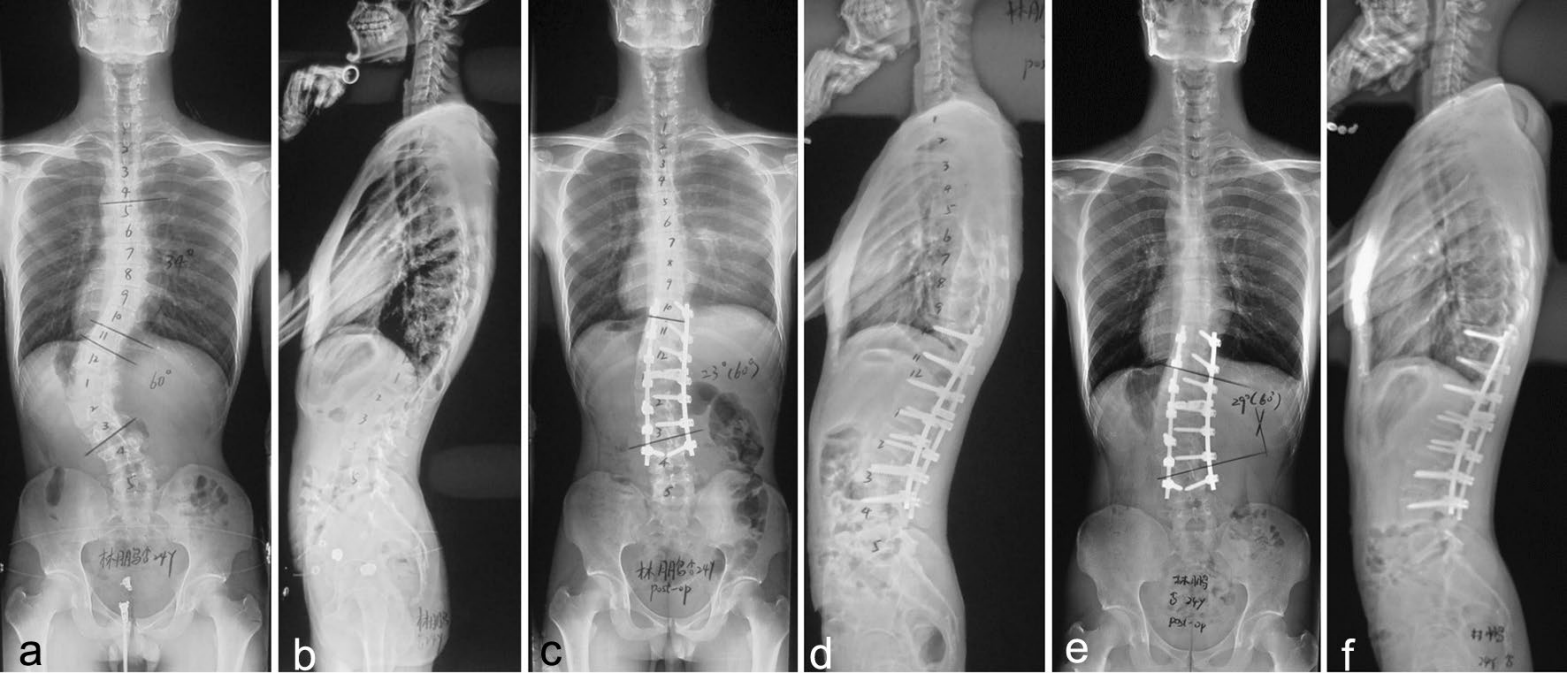
An male patient aged 25 years with 60° left thoracolumbar curve (**a**, **b**), showing greater L3 and L4 translation and thoracolumbar kyphosis of 32°. After T10-L4 posterior correction surgery, thoracolumbar curve was corrected to 23°, and thoracolumbar kyphosis was corrected to 8° (**c**, **d**); 4 year postoperative radiographs did not show significant correction loss (**e**, **f**)

AdIS group had larger preoperative T10-L2 angle (12.6 ± 7.2 vs. 3.2 ± 4.6°; *p* < 0.001) and smaller T5-T12 angle (18.5 ± 11.5 vs. 24.6 ± 13.3°; *p* = 0.024). There was no difference as to L1-S1 angle, pelvic tilt, sacral slope, and pelvic incidence (*P* > 0.05). After the surgery, sagittal parameters have been corrected to normal range and there was no difference as to T10-L2 angle, T5–T12 angle, L1-S1 angle, SVA, pelvic tilt, sacral slope, and pelvic incidence in AdIS and AIS group (*P* > 0.05) (Fig. 2).

CT measurements demonstrated no difference of vertebral body rotation at apical vertebrae and lowest instrumented vertebrae preoperative (*P* > 0.05). However, after the derotation operation, AdIS group still had an extent of vertebral body rotation, while these in AIS group had been corrected to normal range. At last follow-up, the vertebral body rotation at apical vertebrae and lowest instrumented vertebrae were significant severe in the AdIS group (17.5 ± 4.2 vs. 10.4 ± 3.7°, *p* = 0.008 and 14.4 ± 3.4 vs. 9.6 ± 3.6°, *p* = 0.011).

AdIS group had higher preoperative VAS scores (3.2 ± 0.2 vs. 1.2 ± 0.1; *P* < 0.001) and higher pain scores of SRS-22 (3.2 ± 0.3 vs. 4.5 ± 0.2; *P* < 0.001) as compared to AIS group. And there were no significant difference in the rest of the SRS-22 domain (*P* > 0.05). Immediately after the surgery, six patients in AdIS and ten patients in AIS group reported worse pain, which were relieved at final follow-up. There was no difference of VAS scores and SRS-22 scores between the two groups at final follow-up (*P* > 0.05).

Correlation analysis demonstrated positive relationship between VAS scores and T10-L2 angle (*r* = 0.492, *P* < 0.05). There was no difference of correction loss of major curve between AdIS group and AIS group (2.7 VS. 3.1°, *P* > 0.05). Adding-on occurred at 5 patients at AdIS group and 9 at AIS group, and the rate of adding-on was similar between the two groups (*P* > 0.05) (Tables 1, 2 and 3).

**Table 1:** Comparison of preoperative clinical and radiographic parameters between AdIS and AIS

	AdIS (22)	AIS (44)	*P*
Age (year)	37.3 ± 8.7	14.7 ± 3.2	< 0.001
Sex (male:female)	6: 16	15: 29	0.465
Major curve angle (°)	51.5 ± 6.4	49.2 ± 7.3	0.873
T5-T12 angle (°)	18.5 ± 11.5	24.6 ± 13.3	0.024
T10-L2 angle (°)	12.6 ± 7.2	3.2 ± 4.6	< 0.001
L1-S1 angle (°)	47.2 ± 8.7	49.1 ± 9.6	0.672
PI (°)	42.7 ± 10.4	41.7 ± 12.0	0.781
PT (°)	5.3 ± 8.3	4.7 ± 6.9	0.371
SS (°)	37.4 ± 5.7	37.0 ± 5.2	0.628
L3 tilt (°)	29.4 ± 6.4	21.7 ± 5.3	0.027
L4 tilt (°)	23.7 ± 5.7	15.2 ± 4.6	0.017
L3 translation (mm)	33.4 ± 5.6	23.1 ± 4.2	0.012
L4 translation (mm)	22.6 ± 5.7	14.9 ± 5.0	0.021
Rotation of apical vertebra (°)	24.7 ± 7.2	22.3 ± 6.1	0.562
Rotation of LIV (°)	17.2 ± 4.7	14.7 ± 5.6	0.672

**Table 2:** Comparison of final follow-up clinical and radiographic parameters between AdIS and AIS

	AdIS (22)	AIS (44)	*P*
Major curve angle (°)	17.4 ± 8.9	10.2 ± 7.2	0.009
Correction ratio (%)	65.6 ± 15.7	79.1% ± 13.2	0.012
T5-T12 angle (°)	31.6 ± 13.6	33.9 ± 12.8	0.692
T10-L2 angle (°)	2.1 ± 3.2	3.9 ± 3.1	0.198
L1-S1 angle (°)	46.8 ± 10.4	49.2 ± 9.5	0.719
PI (°)	43.1 ± 9.7	42.9 ± 10.1	0.733
PT (°)	9.2 ± 8.4	10.6 ± 7.9	0.561
SS (°)	33.9 ± 7.3	32.3 ± 5.4	0.792
L3 tilt (°)	9.4 ± 4.1	4.2 ± 3.5	< 0.001
L4 tilt (°)	7.2 ± 3.7	3.8 ± 3.2	< 0.001
L3 translation (mm)	22.4 ± 4.3	15.2 ± 4.6	0.029
L4 translation (mm)	16.2 ± 3.9	11.4 ± 3.2	0.016
Rotation of apical vertebra (°)	17.5 ± 4.2	10.4 ± 3.7	0.008
Rotation of LIV (°)	14.4 ± 3.4	9.6 ± 3.6	0.011
Fusion levels	7.8 ± 2.4	6.4 ± 1.6	0.041
Operation time (min)	173.6 ± 32.8	234.2 ± 44.5	< 0.001
Blood loss (ml)	578.3 ± 94.7	739.6 ± 127.3	< 0.001

**Table 3:** Comparison of VAS scores and SRS-22 scores between AIS and AdIS preoperative and at final follow-up

		Preoperative	Final follow-up	*p* value
Pain	AIS	4.5 ± 0.2	4.6 ± 0.2	0.199
	AdIS	3.2 ± 0.3	4.5 ± 0.1	< 0.001
	*p* value	< 0.001	0.211	
Self-image	AIS	3.3 ± 0.2	4.7 ± 0.1	< 0.001
	AdIS	3.1 ± 0.3	4.5 ± 0.2	< 0.001
	*p* value	0.711	0.167	
Psychologic status	AIS	3.4 ± 0.2	4.6 ± 0.2	< 0.001
	AdIS	3.7 ± 0.3	4.5 ± 0.1	< 0.001
	*p* value	0.670	0.188	
Physical function	AIS	4.3 ± 0.2	4.4 ± 0.2	0.333
	AdIS	4.4 ± 0.3	4.4 ± 0.1	0.276
	*p* value	0.311	0.412	
VAS score	AIS	1.2 ± 0.1	0.8 ± 0.2	0.467
	AdIS	3.2 ± 0.2	1.2 ± 0.3	< 0.001
	*p* value	< 0.001	0.422	

## Discussion

From classification aspects, AdIS belongs to adult spine deformity (ASD). ASD includes a broad spectrum of spine deformities, and the current classifications of ASD are mostly descriptive rather than decision-making guided [12]. Lenke classification may be the only one offering guidelines of surgical strategies for AIS [13]. Most surgeons refer to Lenke classification when making surgical strategies in the treatment of AdIS. However, AdIS was different from AIS in many aspects. First, AIS patients always seek correction surgery for psychosocial reasons or halting curve progression, while quite a number of AdIS patients receive surgery for the sake of curing back pain. Watanabe investigated the middle-aged patients with non-surgically treated AIS and compared the health-related quality-of-life (HRQOL) status of different curve types. He proposed that when compared patients with structural thoracolumbar/lumbar curves or patients with single main thoracic curve with the healthy controls, the patients with structural thoracolumbar/lumbar curves are more likely to experience greater annual TL/L curve progression and have substantial or worse low back pain-specific HRQOL status than the healthy controls, while patients with single thoracic curve showed no significant difference with the healthy controls [14]. Fekete investigated whether in patients with AIS and notable back pain surgery was associated with significant pain relief and whether age influences outcome, and found that in patients undergoing surgery for correction of AIS, back pain is correlated with age [15]. In our study, AdIS patients had significantly higher incidence and intensity of back pain than AIS patients with posterior approach. One of the origins of back pain could be thoracolumbar junctional kyphosis. We found significantly larger thoracolumbar junctional kyphosis in AdIS patients than in AIS patients. In addition, degrees of thoracolumbar junctional kyphosis were positively correlated to VAS scores in both AIS patients and AdIS patients. An interesting finding was that worsen pain was noted both in AdIS and AIS patients immediately after surgery; however, at last follow-up, pain was significantly reduced in both groups. Immediately worsen pain might be attributed to muscle stretching after scoliosis correction especially in concave side. Long-term follow-up demonstrated helpfulness of correction surgery for pain alleviation for scoliosis patients coinciding with Helenius’s finding that patients who underwent posterior spinal fusion with pedicle screws experienced improved back pain and health-related quality of life compared with patients with untreated AIS [16].

For Lenke 5c AIS, the choosing of touched vertebrae, neutral vertebrae, or stable vertebrae lacks in-depth discussion. We generally choose the most tilt vertebrae as lower end vertebrae. In the long-time follow-up, the effect of this principle is satisfying. Fusion strategy of Lenke 5c AIS adheres to the so-called “cobb-cobb” principle that fuses vertebrae from upper end vertebra to lower end vertebra [17]. However, the premise of using this principle lies in two aspects. First, lower end vertebra is not far from CSVL. Shu found that horizontalization of the LIV and minimizing LIV translation during correction could reduce the risk of distal adding-on [18]. In a meta-analysis, Yang evaluated the incidence, characteristics, and risk factors for "adding-on", and demonstrated that lowest instrumented vertebra (LIV)-center Sacral Vertical Line (CSVL) and deviation of LIV + 1 were found to be significantly associated with "adding-on" [19]. In the present study, AdIS group had greater preoperative L3 and L4 translation than AIS group. AdIS group had more patients instrumented to L4, even at L5. After operation, L3 and L4 translations were still greater in AdIS group. Second, lower end vertebra should be well derotated. Shu also demonstrated that derotation of the presumed LIV on SB films may hint less risk of distal adding-on in Lenke 5c patients [18]. In another word, well derotation could reduce the incidence of adding-on. CT scans showed both similar preoperative rotations of apical vertebra and LIV between the two groups with posterior approach. After operation, AdIS group achieved less derotation than AIS, which could be attributed to degeneration of facet joints and less elicitability of discs. Similarly, correction ratio was also better in AIS group. As has been stated, AdIS patients had significantly larger thoracolumbar junctional kyphosis than AIS patients, which was one of the treatment goals for AdIS. Postoperative radiographs demonstrated good correction of TJK in AdIS group, which was similar to AIS.

Unsurprisingly, AdIS group had longer fusion level, more operation time, and more blood loss than AIS group. AdIS patients always have severer facet joint degeneration, and releasing facet joint would cost more time and produce greater blood loss. In addition, longer fusion level and stronger muscle would also contribute to prolonged operation time and greater blood loss.

Incidence of adding-on was similar between the two groups. Shu reported an incidence of 18.8% of adding-on in Lenke 5c AIS patients [18]. Ilharreborde reported adding-on in 10.3% AIS patients, of which 62.5% had LIV above last touching vertebra. For most Lenke 5c patients, LIV was chosen at either L3 or L4 [20]. AdIS had more incidence of LIV at L4, which limited the room for developing adding-on. Furthermore, depletion of growth potential also permitted low chance of adding-on occurrence, as previous study had demonstrated positive correlation between growth potential and adding-on [21].

The disc degeneration in the distal mobile segments is an issue of concern and related to the back pain over time following surgery. According to the previous study, LIV to L4 had the highest risk of developing significant disc degeneration. Lonner proposed that avoid fusing to L4, maintain the LIV tilt below 5° and LIV translation less than 2 cm could decrease the incidence of disc degeneration [22]. In our study, though AdIS had more incidence of LIV at L4, the disc degeneration showed no significant difference between AdIS and AIS between two groups at final follow-up. The reason for this phenomenon could be insufficient follow-up time and studies with long follow-up time need to be carried out.

In conclusion, Lenke 5c AdIS patients had greater preoperative and postoperative L3 and L4 tilt and translation, as well as less correction of major curve and vertebral body derotation than AIS patients with posterior approach. However, the incidence of adding-on was similar between the two groups.

## Data Availability

All datasets generated or analyzed during this study are available from the corresponding author on reasonable request.
